# Adipokine Profiling in Adult Women With Central Obesity and Hypertension

**DOI:** 10.3389/fphys.2018.00294

**Published:** 2018-03-27

**Authors:** Rashmi Supriya, Benjamin Y. Yung, Angus P. Yu, Paul H. Lee, Christopher W. Lai, Kenneth K. Cheng, Suk Y. Yau, Lawrence W. C. Chan, Sinead Sheridan, Parco M. Siu

**Affiliations:** ^1^Department of Health Technology and Informatics, Hong Kong Polytechnic University, Kowloon, Hong Kong; ^2^School of Public Health, Li Ka Shing Faculty of Medicine, University of Hong Kong, Pokfulam, Hong Kong; ^3^School of Nursing, Hong Kong Polytechnic University, Kowloon, Hong Kong; ^4^Department of Rehabilitation Sciences, Faculty of Health and Social Sciences, Hong Kong Polytechnic University, Kowloon, Hong Kong

**Keywords:** high blood pressure, abdominal obesity, inflammation, adipocyte, diabetes, stroke, coronary artery disease, renal disease

## Abstract

Central obesity and hypertension are common risk factors for the metabolic syndrome, cardiovascular and renal diseases. Studies have shown that it is more difficult to control blood pressure and prevent end-organ damage in obese individuals with hypertension compared to their non-obese counterparts, especially among women. Obese females have a 6 times higher risk of developing hypertension than non-obese females while obese males are at a 1.5 times higher risk of developing hypertension, compared to their non-obese counterparts. Indeed, the inter-relationship between obesity and hypertension is unclear. Adipokines have been proposed to play a mediating role in the relationship between obesity and hypertension and are involved in the pathogenesis of metabolic diseases. Therefore, this study sought to determine the role of adipokines (adiponectin, plasminogen activator inhibitor-1, leptin, and tumor necrosis factor-α) in hypertensive Hong Kong Chinese women with central obesity. A total of 387 women aged 58 ± 11 years who were examined with a 2 × 2 factorial design for central obesity (waist circumference ≥ 80 cm) and hypertension (blood pressure ≥ 140/90 mmHg), were recruited from a pool of 1,492 Hong Kong Chinese adults who were previously screened for metabolic syndrome. Subjects with hyperglycemia, hypertriglyceridemia, and dyslipidemia were excluded to eliminate confounding effects. Our findings revealed that hypertensive women with central obesity had a lower anti-inflammatory status (adiponectin) and a higher pro-inflammatory status (TNF-α) than obese alone or hypertensive alone women. Also, women with central obesity had higher circulatory PAI-1 and leptin concentrations than their non-obese counterparts. We conclude that obesity may shift toward a more pro-inflammatory state and may become more severe in the presence of hypertension or vice versa.

## Introduction

Obesity and hypertension are commonly associated with chronic disorders, including metabolic syndrome (MetS), renal disease, stroke, and cardiovascular diseases (Hall, 2000; Frohlich, 2002; Hall et al., 2002; Wofford and Hall, 2004). According to the obesity paradox, lean individuals with hypertension have lower survival rates than obese individuals (Barrett-Connor and Khaw, 1985; Goldbourt et al., 1987). On the other hand, obese hypertensive individuals have a higher mortality rate and have an increased risk of cardiovascular diseases compared with non-obese hypertensive individuals (Chiang et al., 1969; Messerli, 1982). Indeed, the inter-relationship between obesity and hypertension is unclear. Limited studies have sought to examine the subtle differences between the sequelae observed in lean individuals with hypertension compared to obese counterparts (Chiang et al., 1969; Frohlich, 2002). Obese individuals have been reported to develop more cardiovascular structural abnormalities, whereas those obese individuals with hypertension have an increased risk of renal insufficiency (Hall, 2000; Frohlich, 2002; Hall et al., 2002; Wofford and Hall, 2004; Lavie et al., 2007). It has been hypothesized that the higher death rate observed in obese hypertensive individuals compared to obese alone individuals may be the result of a combination of several overlapping factors, including the regulation of appetite and satiety, endothelial function, energy expenditure, haemostasis, insulin sensitivity, blood pressure, adipogenesis, fat distribution, and insulin secretion in pancreatic β-cells, all of which have been attributed to obesity and hypertension (Re, 2009).

Over the past number of years, the majority of studies have focused on examining the differences between obese hypertensive individuals and lean normotensive individuals or obese normotensive individuals (Dustan, 1990; Philip-Couderc et al., 2004; Re, 2009). For example, it has been shown that it is more difficult to control blood pressure and end-organ damage in obese individuals with hypertension compared to their non-obese counterparts, especially in females (Gudmundsdottir et al., 2012; Jesky et al., 2015). The risk of hypertension is higher in females compared to males, with obesity reported as the most substantial risk factor (Mosca et al., 2011). Thus, the inclusive healthcare burden of obesity-associated hypertension in females is likely surpasses that of males. Several mechanisms, including neurohumoral pathways, the function of adipose tissue derivatives (adipokines and cytokines), metabolic functions and the modulation of pressor/depressor mechanisms, have all been proposed (Kotsis et al., 2010). Stimulation of the renin-angiotensin system (RAS) has been reported as one of the essential mechanisms in obesity-related hypertension (Wofford et al., 2001; Frohlich, 2002). In obesity, exacerbation of the RAS components of adipocytes leads to systemic RAS consequences (Wofford and Hall, 2004), including dysregulation of renal vasomotor activity, uncontrolled tissue growth in the kidneys, and disruption of optimal salt and water homeostasis (Brewster and Perazella, 2004). Diet-induced obesity upregulates RAS in adipose tissue that increases reactive oxygen species, elevates lipogenesis, impairs insulin signaling, and promotes inflammation in the circulation (Wofford et al., 2001; Frohlich, 2002; Wofford and Hall, 2004). Thus, the activation of RAS in the circulation represents a link between inflammation during obesity and hypertension (Wofford and Hall, 2004).

Intriguingly, adipokines have been shown to be associated with RAS. Adipose tissue is a large endocrine organ, which secretes biologically active substances called adipokines, such as adiponectin, plasminogen activator inhibitor-1 (PAI-1), tumor necrosis factor-α (TNF-α), and leptin (Wiecek et al., 2002). A RAS blockade by an angiotensin-converting enzyme inhibitor and angiotensin II receptor blocker has been demonstrated to increase plasma adiponectin in individuals with MetS (Tian et al., 2010). Leptin has been reported to upregulate RAS and pro-inflammatory cytokines, as well as to mediate angiotensin II-elicited hypertension in rats fed a high-fat diet for 3 weeks (Xue et al., 2016). Furthermore, an increase in PAI-1, which is a pro-inflammatory adipokine involved in the coagulation system, is associated with RAS and cardiovascular risk factors, such as hypertension, obesity, insulin resistance and diabetes (Ploplis, 2011; Raiko et al., 2012). TNF-α, which is a well-known pro-inflammatory cytokine with a wide range of biological effects, has also been reported to be involved in functional crosstalk with angiotensin II that causes adverse left ventricular remodeling and hypertrophy in hypertension (Sriramula and Francis, 2015).

The inter-relationship between obesity and hypertension is unclear and the role of adipokines remains to be elucidated in hypertensive female adults with obesity. Therefore, this study was designed to determine the role of adipokines (adiponectin, plasminogen activator inhibitor-1, leptin, and TNF-α) in hypertensive Hong Kong Chinese women with established central obesity. We hypothesized that the interaction between central obesity and hypertension would exacerbate the adipokine equilibrium by upregulating pro-inflammatory adipokines and downregulating anti-inflammatory adipokine in adult women.

## Materials and methods

### Study design

This is a cross-sectional study in which blood samples from a total of 387 women (mean age ± *SD* = 58 ± 11 years) were selected from a pool of 1,492 Hong Kong Chinese adults who were previously screened for MetS using the United States National Cholesterol Education Program (NCEP) Expert Panel Adult Treatment Panel (ATP) III guidelines (Siu et al., 2015). Subjects diagnosed with MetS had more than two of following characteristics: (1) central obesity (waist circumference exceeds 90 or 80 cm for Asian male and female, respectively), (2) hypertension (systolic pressure equals or exceed 130 mmHg, or diastolic pressure equals or exceeds 85 mmHg), (3) elevated blood glucose (fasting glucose level equals or exceeds 5.5 mmol/L [100 mg/dL]), (4) elevated plasma triglycerides (level equals or exceeds 1.70 mmol/L [150 mg/dL]), and (5) low level of HDL-C (level equals or is less than 1.0 mmol/L [40 mg/dL] for male and 1.3 mmol/L [50 mg/dL] for female) were regarded as MetS positive. Participants with neuro-musculoskeletal illness, post-stroke, severe, or acute cardiovascular diseases, dementia or mental disorders, symptomatic heart or lung diseases, acute medical illness, osteoarthritis or pulmonary illness, smoker, severe rheumatoid arthritis, and participants who were under treatment for metabolic abnormalities were excluded from the study (Yu et al., 2015). Subject participation was voluntary and written informed consent was obtained prior to study commencement. Subjects could withdraw from the study before or at any time during the study. Human research ethics approval was granted by the Human Subjects Ethics Subcommittee of Hong Kong Polytechnic University (HSEARS20150205001). All methods were performed in accordance with the relevant guidelines and regulations.

### Subject selection

Female subjects with central obesity (i.e., a waist circumference ≥80 cm based on NCEP ATP III criteria, Siu et al., 2015), with hypertension (i.e., a systolic blood pressure ≥140 mmHg and/or a diastolic blood pressure ≥90 mmHg based on the Seventh Report of the Joint National Committee on Prevention, Detection, Evaluation, and Treatment of High Blood Pressure, Chobanian, 2003) and with central obesity and hypertension were selected for the study. Subjects with a high fasting blood glucose of ≥5.5 mmol/L, high plasma triglycerides ≥1.70 mmol/L and low high-density lipoprotein (HDL) cholesterol (HDL-C ≤ 1.3 mmol/L were excluded. This study design allowed us to supply an unambiguous interpretation solely based on the individual and interaction effects of central obesity and hypertension, not confounded by other MetS risk factors, including hyperglycemia, hypertriglyceridemia, and reduced HDL cholesterol (Table 1). Subjects were divided into four groups: (1) Non-central obese subjects with normal blood pressure (NO_NBP; *n* = 105), (2) Non-central obese subjects with hypertension (NO_HBP; *n* = 102), (3) Central obese subjects with normal blood pressure (O_NBP; *n* = 74), and (4) Central obese subjects with hypertension (O_HBP; *n* = 106).

**Table 1:** Baseline characteristics of age and metabolic risk factors in the following 4 groups: (1) Non-central obese subjects with normal blood pressure (NO_NBP; *n* = 105), (2) Non-central obese subjects with hypertension (NO_HBP; *n* = 102), (3) Central obese subjects with normal blood pressure (O_NBP; *n* = 74), and (4) Central obese subjects with hypertension (O_HBP; *n* = 106).

	**Group 1 (NO_NBP) *N* = 105**	**Group 2 (NO_HBP) *N* = 102**	**Group 3 (O_NBP) *N* = 74**	**Group 4 (O_HBP) *N* = 106**
Age (Years)	50 ± 5	64 ± 10	50 ± 6	63 ± 10
Diastolic blood pressure (mmHg)	68.6 ± 6.3	79.8 ± 11.2	71.8 ± 7.2	81.5 ± 11.6
Systolic blood pressure (mmHg)	111.5 ± 9.7	161.3 ± 14.2	115.5 ± 8.2	158.4 ± 14.8
Waist circumference (cm)	72.6 ± 4.2	73.3 ± 4.6	85.9 ± 4.8	87.1 ± 5.6
Fasting glucose (mmol/L)	4.8 ± 0.3	4.9 ± 0.3	4.8 ± 0.3	4.9 ± 0.3
Blood triglycerides (mmol/L)	1.0 ± 0.3	0.9 ± 0.3	1.0 ± 0.3	1.0 ± 0.2
Blood high density lipoprotein-C (mmol/L)	1.7 ± 0.3	1.7 ± 0.3	1.7 ± 0.2	1.6 ± 0.3

*The data are expressed as the mean ± standard deviation*.

### Cardiometabolic risk factors of metabolic syndrome

All the MetS diagnostic parameters were measured by trained personnel. An electronic blood pressure monitor (Accutorr Plus, Datascope) was used to measure systolic, and diastolic blood pressure over the brachial artery region on the right arm supported at heart level after 5 min of rest with the use of appropriately sized cuffs. The inelastic measuring tape was used on the open skin region between the lowest rib and superior border of the iliac crest to measure waist circumference. Venous blood samples were obtained by certified phlebotomists from subjects after at least 10 h of fasting. An automatic clinical chemistry analyzer (Architect CI8200, Abbott Diagnostics) in an accredited medical laboratory was used to determine fasting blood glucose, blood HDL-cholesterol, and blood triglycerides.

### Adipokines and insulin

Commercially available enzyme-linked immunosorbent assay (ELISA) kits were used to perform biochemical measurements for adipokines and insulin in the harvested serum samples according to the manufacturer's instructions. Adiponectin, PAI-1, leptin and TNF-α ELISA kits were purchased from R&D, and insulin kits were purchased from Thermo Fisher Scientific. The coefficient of variability (CV) for the ELISA kits were adiponectin (intra-assay: 3.5%; inter-assay: 7%), PAI-1 (intra-assay: 6.8%; inter-assay: 7%), leptin (intra-assay: 3%; inter-assay: 4.4%) and TNF-α (intra-assay: 4.9–7.8%; inter-assay: 4.7–5.8%). Measurements were performed in duplicates or triplicates by a single observer to minimize the observer variation.

### Statistical analysis

Data was expressed as the mean ± standard deviation. The generalized estimating equation was adopted to analyze the non-normal distribution of the data where the adipokines were considered as dependent variables. Central obesity and hypertension were considered as the two independent factors with binary traits. Interaction effect was analyzed by adjusting fasting glucose level, triglycerides level, high-density lipoprotein level and age as covariates. Sensitivity analysis was performed for calculating interaction between waist circumference and blood pressure (BP) systolic and diastolic as independent factors with continuous traits on adipokine levels. A Kruskal-Wallis test followed by a *post hoc* test with a Dunn-Bonferroni correction were used to investigate multiple group-wise comparisons. All statistical analyses were performed using the Statistical Package for the Social Sciences (SPSS) version 22 for Windows. Data are expressed as the mean ± standard deviation. Statistical significance was accepted at *P* < 0.008 (i.e., *P* value/total number of comparisons = 0.05 / {(4 × 3)/2}) to minimize the possibility of false positive results.

## Results

### Circulatory levels of TNF- α were higher, and adiponectin were lower in obese hypertensive women than in women with obesity alone or hypertension alone

**Figure 1:**
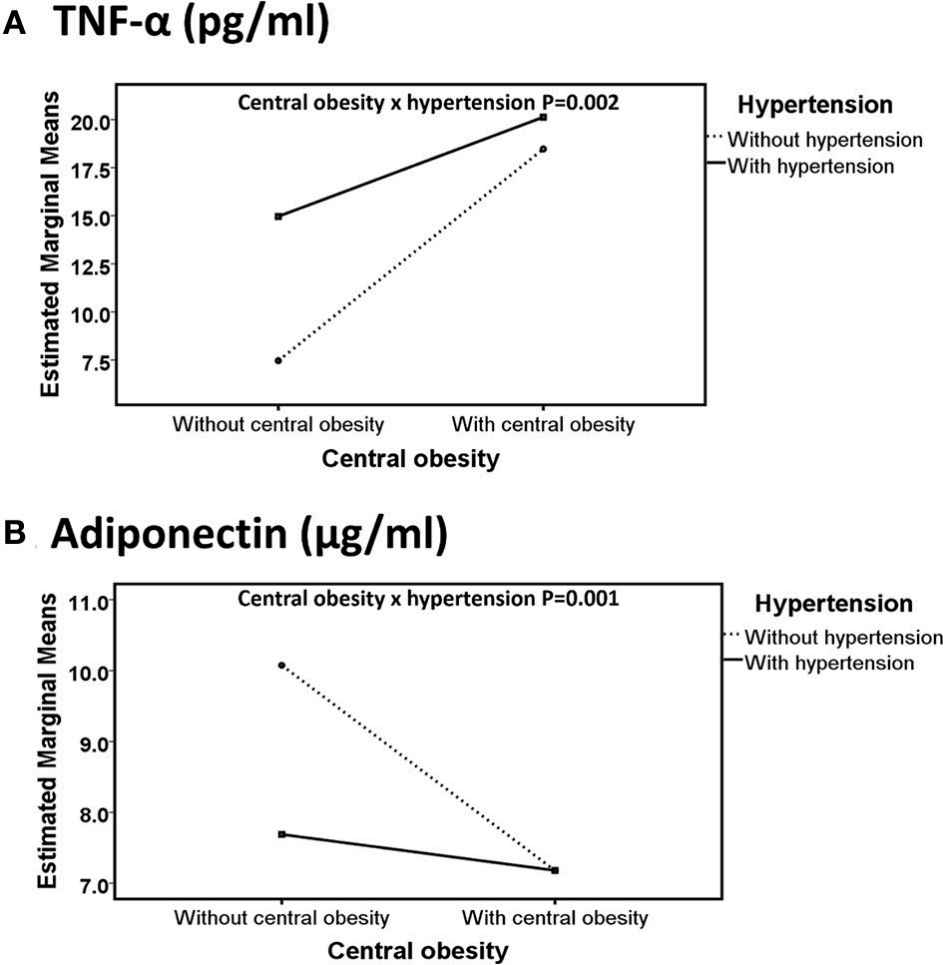
Line graphs represent the directions of interaction effect of central obesity and hypertension on adipokines including TNF-α **(A)** and adiponectin **(B)** in Hong Kong Chinese women categorized into four groups, including (1) Non-central obese subjects with normal blood pressure (NO_NBP; *n* = 105), (2) Non-central obese subjects with hypertension (NO_HBP; *n* = 102), (3) Central obese subjects with normal blood pressure (O_NBP; *n* = 74), and (4) Central obese subjects with hypertension (O_HBP; *n* = 106). Subjects with hypertension were defined as having systolic blood pressure ≥140 mmHg and diastolic blood pressure ≥90 mmHg, and subjects with central obesity were defined as having a waist circumference ≥80 cm. The data are expressed in estimated marginal means.

Interaction effects of central obesity and hypertension with binary traits were found to affect TNF-α (Wald chi square = 10.1, *P* = 0.002; Figure 1A) and adiponectin (Wald chi square = 10.3, *P* = 0.001; Figure 1B). Compared with non-obese normotensive subjects, mean difference of adiponectin in obese normotensive subjects was 2.9 μg/ml (*P* < 0.001) and did not appear to differ from central obese hypertensive subjects. Compared with non-obese normotensive subjects, mean difference of adiponectin in non-obese hypertensive subjects was 2.3 μg/ml (*P* < 0.001) and was lower than in central obese hypertensive subjects (Figure 1A). Compared with non-obese and normotensive subjects, mean difference of TNF-α in obese normotensive and non-obese hypertensive subjects were −11.01 pg/ml and −7.50 pg/ml (*P* < 0.001) which were lower than in central obese hypertensive subjects −12.67 pg/ml (*P* < 0.001) (Figure 1B).

After adjusting for fasting glucose level (mean ± *SD* = 88.09 ± 6.06 mg/dl), triglycerides (mean ± *SD* = 90.96 ± 25.08 mg/dl) and high-density lipoprotein (mean ± *SD* = 65.3 ± 11.4 mg/dl) and age (mean = 57) as covariates, the interaction effect was still significant for both adiponectin and TNF- α. Sensitivity analysis for interaction between waist circumference and BP systolic and diastolic as independent factors with continuous traits were only found on TNF-α (Supplementary Table 1). Sensitivity analysis for interaction between waist circumference BP systolic were also found on adiponectin and PAI-1; and, interaction between waist circumference and BP diastolic was found on leptin (Supplementary Table 1).

**Figure 2:**
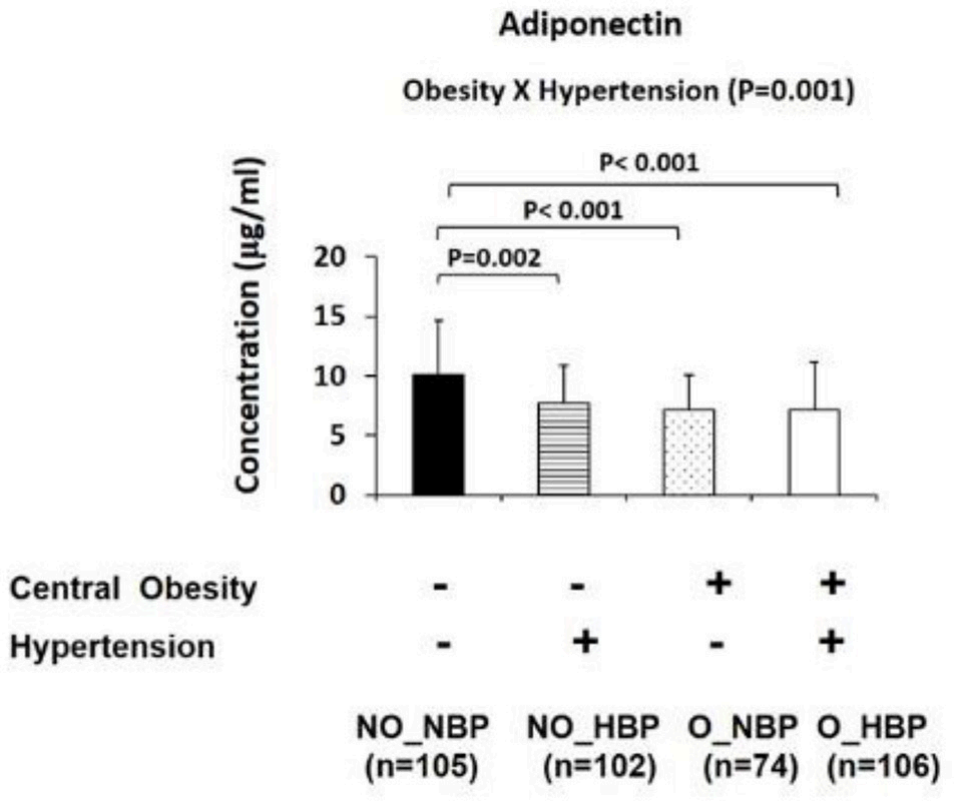
Bar graphs represent circulatory abundance of anti-inflammatory adipokine adiponectin in Hong Kong Chinese women categorized into four groups, including (1) Non-central obese subjects with normal blood pressure (NO_NBP; *n* = 105), (2) Non-central obese subjects with hypertension (NO_HBP; *n* = 102), (3) Central obese subjects with normal blood pressure (O_NBP; *n* = 74), and (4) Central obese subjects with hypertension (O_HBP; *n* = 106). Subjects with hypertension were defined as having systolic blood pressure ≥140 mmHg and diastolic blood pressure ≥90 mmHg, and subjects with central obesity were defined as having a waist circumference ≥80 cm. The data are expressed as the mean ± standard deviation.

**Figure 3:**
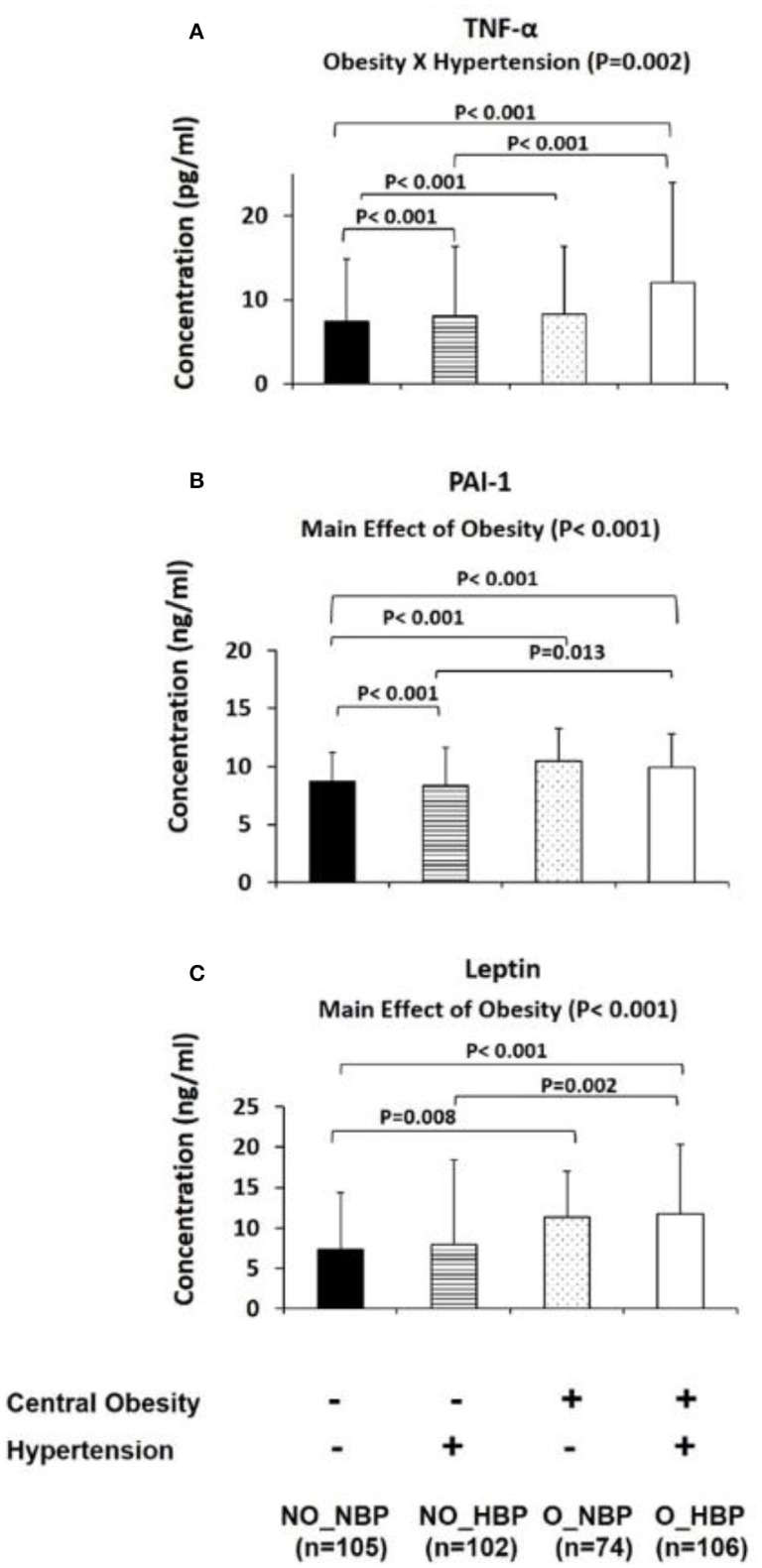
Bar graphs represent circulatory abundance of pro-inflammatory adipokines, including PAI-1 **(A)**, TNF-α **(B)**, and leptin **(C)** in Hong Kong Chinese women categorized into four groups, including (1) Non-central obese subjects with normal blood pressure (NO_NBP; *n* = 105), (2) Non-central obese subjects with hypertension (NO_HBP; *n* = 102), (3) Central obese subjects with normal blood pressure (O_NBP; *n* = 74), and (4) Central obese subjects with hypertension (O_HBP; *n* = 106). Subjects with hypertension were defined with systolic blood pressure ≥140 mmHg and diastolic blood pressure ≥90 mmHg, and subjects with central obesity were defined as having a waist circumference ≥80 cm. The data are expressed as the mean ± standard deviation.

Furthermore, the four group comparisons indicated that NO_NBP had a 23.8% (*P* = 0.002) higher adiponectin concentration and 98.7% (*P* < 0.001) lower TNF-α concentration than NO_HBP (i.e., the difference between normotensive and hypertensive in non-obese subjects). NO_NBP had a 28.7% (*P* < 0.001) higher concentration and a 146% (*P* < 0.001) lower TNF-α concentration compared with O_NBP (i.e., the difference between non-obese and central obese in normotensive subjects). NO_NBP had a 28.7% (*P* < 0.001) higher adiponectin concentration and a 168% (*P* < 0.001) lower TNF-α concentration than O_HBP (i.e., the difference between non-obese normotensive and central obese hypertensive subjects). NO_HBP had a 34.4% (*P* < 0.001) lower TNF-α concentration compared with O_HBP (i.e., the difference between non-obese and central obese in hypertensive subjects) (Figures 2, 3A).

### Circulatory levels of leptin and PAI-1 were higher in central obese women than non-obese women

The main effects of central obesity were observed on PAI-1 (Wald chi-square = 31.7, *P* < 0.001; Figure 3B) and leptin (Wald chi-square = 21.1, *P* < 0.001; Figure 3C). The means for PAI-1 and leptin were lower in non-central obese subjects (8.52 ± 0.18 ng/ml and 7.61 ± 0.44 ng/ml, respectively) compared with central obese subjects (10.19 ± 0.23 ng/ml and 11.53 ± 0.73 ng/ml, respectively) (Figures 3B,C).

## Discussion

Obesity and hypertension are commonly associated with chronic disorders, including MetS, renal disease, stroke, and cardiovascular diseases (Hall, 2000; Frohlich, 2002; Hall et al., 2002; Wofford and Hall, 2004). Adipokines have been proposed as the link between obesity and hypertension and are related to the pathogenesis of metabolic diseases. Previous researches have shown that TNF-α gene locus contributes to the pathogenesis of obesity and obesity-associated hypertension both in males and females (Pausova et al., 2000). Our results indicated that the concentration of pro-inflammatory adipokine TNF-α was higher in hypertensive women with central obesity than hypertensive or obese alone women. Dreier et al. reported that obese hypertensive males have similar adiponectin concentrations as obese normotensive males (Dreier et al., 2016). In contrast, our findings demonstrated that the concentration of anti-inflammatory adipokines (adiponectin) was lower in hypertensive adult women with central obesity. Therefore, according to our results, gender-specific treatment for obesity and hypertension may be required due to the differences in cardiovascular physiology between sexes (Huxley, 2006). Our findings are specific to adult women and support that obesity-related hypertension is disadvantageous as it leads to a shift toward a more pro-inflammatory state compared with central obesity or hypertension alone.

Our results showed that central obesity and hypertension interacted to affect circulatory concentration of adiponectin and TNF-α. Compared with non-obese normotensive subjects, the decrease in circulatory adiponectin in hypertensive-alone subjects was relatively smaller than obese hypertensive and obese-alone subjects (Figure 1A). Similarly, a study reported that hypertensive individuals had significantly lower circulatory adiponectin compared with body mass index-matched normotensive healthy individuals (9.1 ± 4.5 vs. 13.7 ± 5.2 μg/mL; Adamczak, 2003). Furthermore, we found an interaction effect between central obesity and hypertension on circulatory TNF-α concentration. The increase in circulatory TNF-α concentration in hypertensive-alone subjects and in central obese-alone subjects was relatively smaller than obese hypertensive subjects, compared with non-obese normotensive subjects (Figure 1B). Adipokines and obesity in women is associated with renal complications or cardiovascular diseases by mediating endothelial dysfunction, oxidative stress, inflammation and changes in immune response (Kotsis et al., 2010). Therefore, diminution of adiponectin (an anti-inflammatory adipokine) and exacerbation of TNF-α (a proinflammatory adipokine) in obese hypertensive subjects may be associated with severe metabolic disorders such as adverse cardiovascular or renal diseases.

### Higher circulatory TNF-α and lower adiponectin levels in obese hypertensive women than hypertensive or obese-alone women may be associated with an increased risk of developing cardiometabolic diseases

The mechanisms through which central obesity interacts with hypertension are mostly unknown. Some mechanisms underlying the pathogenesis of obesity-related hypertension were proposed based on studies in humans and animals, and such mechanisms include neurohumoral pathways and adipose-tissue derivatives (adipokines and cytokines) in both functional and metabolic mechanisms. Circulating adiponectin concentration has been shown to be negatively associated with pro-inflammatory cytokines, such as IL-6 and TNF-α (Esposito et al., 2003; Krakoff et al., 2003). Adiponectin exhibits anti-inflammatory action through multiple mechanisms by inducing changes in phenotype and function of macrophages (Yamaguchi et al., 2005). The increase in adiponectin has also been shown to inhibit the expression of adhesion molecules and to suppress the adherence of TNF-α-stimulated endothelial cells to monocytes (Ouchi et al., 1999). Interestingly, adiponectin has been shown to decrease the agonist-stimulated production of TNF-α in cultured macrophages, which was accompanied by reduced nuclear factor-κB (NFκB) activation (Yamaguchi et al., 2005). NFκB is a regulatory molecule that regulates pro-inflammatory cytokines such as TNF-α (Lawrence, 2009). TNF-α is a key pro-inflammatory cytokine associated with the pathology of obesity-linked vascular and metabolic disorders. Cross-talk between angiotensin II and pro-inflammatory adipokines (TNF-α) participates in self-amplifying and sustaining positive feedback loops, which results in the progression of hypertension and cardiac remodeling (Sriramula and Francis, 2015). Mice deficient in the TNF-α gene were found to attenuate the angiotensin-II-induced hypertensive response (Sriramula et al., 2008). Inhibition of TNF-α has also been shown to decrease oxidative stress, NF-κB activation and mitogen-activated protein kinases (MAPK) phosphorylation (Kotsis et al., 2010). Collectively, TNF-α induced hypertension and adverse cardiac remodeling via angiotensin II, were associated with changes in the MAPK/ TGF-β/NF-κB pathway (Kotsis et al., 2010). Therefore, according to our results, the interaction between central obesity and hypertension may lead to a reduction in anti-inflammatory adipokines, adiponectin, and further increases in pro-inflammatory adipokines and TNF-α compared with female subjects with central obesity or hypertension alone. Our results may provide insight for explaining the high mortality rate documented in central obese female with hypertension. Additional research is needed to further determine the exact relationship between our observed interaction and the modulation of adipokines in cardiovascular health. In overweight and obese subjects, the cardiovascular risk is not significantly increased unless hypertension is present (Thomas et al., 2005).

### Higher circulatory TNF-α and lower adiponectin concentration in obese hypertensive women than hypertensive or obese-alone women may be associated with an increased risk of developing renal dysfunction and chronic kidney disease

Activation of the sympathetic nervous system is considered to be an important mechanism underlying the pathogenesis of obesity-related hypertension (Kotsis et al., 2010). In obese individuals, the arterial pressure controlling the function of natriuresis and diuresis shifts toward a higher level of blood pressure. It is proposed that renal tubular reabsorption is increased in the early stages of obesity. This reabsorption results in primary sodium retention that causes the expansion of extracellular fluid volume and the resettlement of the kidney-fluid apparatus to a hypertensive level, which mimics the developmental model of hypertension (Kotsis et al., 2010). Obesity and hypertension are strongly correlated, and both are major risk factors for renal dysfunction and chronic kidney disease. Studies have shown that only specific adipokines are associated with the development of renal dysfunction and chronic kidney disease (Doumatey et al., 2012; Pruijma et al., 2012). Adiponectin has been explored as an independent predictor of moderate chronic kidney disease and is inversely associated with renal dysfunction and chronic kidney disease (Doumatey et al., 2012). The mechanism is still unknown in humans but has been investigated in mice with hypoadiponectinemia and albuminuria (a pathological condition where the protein albumin is abnormally present in the urine) in which a link between hypoadiponectinemia and kidney dysfunction has been demonstrated. Podocytes (specialized cells) are present in the Bowman's capsule in the kidneys. Podocytes wrap around glomerulus capillaries and form multiple interdigitating foot processes (Drumond et al., 1994; Kriz et al., 1994; Briffa et al., 2013). Adiponectin treatment in mice with albuminuria improved the glomerular podocyte foot processes by activating 5′ adenosine monophosphate-activated protein kinase (AMPK). This activated AMPK downregulated nicotinamide adenine dinucleotide phosphate oxidase production in the podocytes (Sharma et al., 2008). Apart from adiponectin, TNF-α has also been associated with the development of renal dysfunction and chronic kidney disease. It is mainly produced by macrophages infiltrating adipose tissue in obesity but can also be produced in the renal cells of the kidney. In the renal cells, TNF-α synthesis can be stimulated by activation of RAS system (Tang et al., 2012). In rats with renal failure, neutralization of TNF-α decreased NFκB activity that was associated with an improvement of nitric oxide released by reduction in renal transforming growth factor beta 1 and endothelin-1 production. Therefore, TNF-α has also been proposed as one of the key adipokines in the progression of renal injury and chronic kidney diseases (Pruijma et al., 2012).

### Higher circulatory concentration of leptin and PAI-1 in central obese women than non-obese women may indicate the risk of hypertension

Our analyses showed significant main effects of central obesity on circulating leptin and PAI-1. Consistent with our results, leptin has been shown to be remarkably upregulated with increased fat mass (Lönnqvist et al., 1995). The circulatory leptin is transported across the blood-brain barrier to a hypothalamic region that controls the transmission of appetite neuropeptides to the peripheral tissues ^45^. The main function of leptin is to stimulate sympathetic activity, upregulate thermogenesis, upregulate energy expenditure, and decrease food consumption (Hall et al., 2000; Wynne, 2005). These effects of leptin are known to be mediated by two main pathways, one driven by a high level of leptin called a positive regulatory action, and the other by an agouti-related peptide, which is an antagonistic ligand activity that stimulates the sympathetic nervous system by activating the hypothalamus-pituitary-adrenal axis (Hall et al., 2000; Wynne, 2005). Interestingly, in anesthetized animals, systemic administration of leptin has been shown to exert no effect on the heart rate or increase in arterial pressure. Therefore, long-term exposure to hyperleptinemia has been suggested for full expression of the expected renal sympathoexcitation pressor effect (Haynes et al., 1997). Nevertheless, leptin has been demonstrated to upregulate RAS and pro-inflammatory cytokines and mediate angiotensin II-elicited hypertension after feeding rats a high-fat diet for 3 weeks (Xue et al., 2016). Therefore, it is plausible that obesity exerts its effect on leptin and that leptin subsequently participates in increasing blood pressure by slowly increasing sympathetic nervous system activity. In addition to leptin, our data also indicate that obesity exerts its effect on PAI-1. Consistent with our findings, increased PAI-1 levels in plasma have been observed among non-diabetic abdominal obese subjects (Vague et al., 1989; Landin et al., 1990). Our result also indicates that there was no significant change in PAI-1 level in hypertensive women. In line with this result, RAS blockade has been shown to slow the progression of processes related to vascular disorders without affecting the PAI-1 level (Baluta and Vintila, 2015). These results may potentially be explained by the involvement of additional pathways, such as decreased fibrinolysis and thrombosis, as well as the interaction between RAS and haemostasis. Therefore, it is possible that obesity exerts its effect on PAI-1 and that PAI-1 subsequently participates in increasing blood pressure by associating with plasma renin activity and insulin resistance (Srikumar et al., 2002).

The cross-sectional design in this study was adopted to infer on the profiles of adipokines in adult women with obesity and hypertension. However, we cannot deduce any casual inference and the results should not be applied to hyperglycemic and dyslipidemic women. Due to the modest sample size, the risks of false negative and false positive results may also be the limitation of the study. In conclusion, our data suggests that the interaction between central obesity and hypertension might lead to the reduction in adiponectin that more than likely exacerbates the TNF-α production and increases the proinflammatory status of adipokines in circulation. These altered adipokine patterns (low circulating adiponectin and high TNF-α) indicate that the effect of obesity might become severe in the presence of hypertension and vice versa. However, the results of this study are specific to adult women, and similar investigation specific in males remains to be explored. Additional research that clarifies the mechanisms concerning adipose tissue derivatives, including adipokines and cytokines, may help to understand and prevent severe outcomes from obesity-related hypertension.

## Author contributions

RS contributed to design, conduct/data collection, analysis of the paper, and writing of the paper. BY contributed to design, analysis of the paper, and writing of the paper. AY contributed to data collection. PL contributed to statistical analyses. CL, LC, KC, SY, and SS contributed to analysis of the paper. PS contributed to the design, analysis of the paper, and writing of the paper.

### Conflict of interest statement

The authors declare that the research was conducted in the absence of any commercial or financial relationships that could be construed as a potential conflict of interest.

## Supplementary material

Supplementary Table 1 Table represents sensitivity analysis for calculating interaction between waist circumference and blood pressure (BP) systolic and diastolic as independent factors with continuous traits on adipokine levels.
