# Supplementary material for: Adipokine Profiling in Adult Women With Central Obesity and Hypertension
Source: Front Physiol. 2018 Mar 27;9:294. doi: 10.3389/fphys.2018.00294 (PMC5881161; doi:10.3389/fphys.2018.00294)
Supplement: Supplementary Table 1 — Table represents sensitivity analysis for calculating interaction between waist circumference and blood pressure (BP) systolic and diastolic as independent factors with continuous traits on adipokine levels. [file Table1.docx]

**Supplementary Table 1**

| **Dependent variables** | **Independent variables**  **(Intercept)** | **Wald chi square** | **P value** |
| --- | --- | --- | --- |
| TNFα | BP_systolic * Waist circumference | 45.66 | < 0.001 |
|  | BP diastolic * Waist circumference | 26.65 | < 0.001 |
| Adiponectin | BP_systolic * Waist circumference | 20.90 | < 0.001 |
|  | BP diastolic * Waist circumference | 0.98 | 0.32 |
| Leptin | BP_systolic * Waist circumference | 3.49 | 0.06 |
|  | BP diastolic * Waist circumference | 11.15 | 0.001 |
| PAI-1 | BP_systolic * Waist circumference | 5.10 | 0.02 |
|  | BP diastolic * Waist circumference | 1.08 | 0.29 |
